# Incidence and predictors of spontaneous thyroid-stimulating hormone normalization after lobectomy for low-risk papillary thyroid microcarcinoma: evidence from contemporary clinical practice

**DOI:** 10.3389/fsurg.2026.1821314

**Published:** 2026-07-06

**Authors:** Zhou Ting Li, Ling Ye, Wu Long Du, Wu Jing Liu, Yan Jie Zhao, Lei Zhu, Jian Song Ji, Feng Cheng

**Affiliations:** 1 Head and Neck Surgery, The Fifth Affiliated Hospital of Wenzhou Medical University, Lishui, China; 2 Department of Ultrasonic Medicine, The Fifth Affiliated Hospital of Wenzhou Medical University, Lishui, China; 3 Lishui Center for Disease Control and Prevention, Lishui, China; 4 Zhejiang Key Laboratory of Imaging and Interventional Medicine, Key Laboratory of Precision Medicine of Lishui City, The Fifth Affiliated Hospital of Wenzhou Medical University, Lishui, China

**Keywords:** levothyroxine supplementation, lobectomy, low-risk papillary thyroid microcarcinoma, spontaneous TSH normalization, thyroid-stimulating hormone

## Abstract

**Objective:**

To investigate spontaneous thyroid-stimulating hormone (TSH) normalization (TSH ≤ 2 μIU/mL) and its predictors in patients with low-risk papillary thyroid microcarcinoma (PTMC) who underwent unilateral lobectomy without pharmacological intervention.

**Methods:**

This retrospective study enrolled 411 low-risk PTMC patients who underwent unilateral lobectomy. Clinical, pathological, and preoperative biochemical data were collected, including thyroid function tests (fT3, fT4, TSH), thyroglobulin (Tg), and thyroid autoantibodies (TPO-Ab, Tg-Ab). TSH levels were monitored at 0.5, 1.5, 6, and 12 months postoperatively. Binary logistic regression and ROC curve analyses were used to identify preoperative predictors of spontaneous TSH normalization at 12 months.

**Results:**

The rate of spontaneous TSH normalization progressively declined: 32.4% at 0.5 months, 20.2% at 1.5 months, 14.8% at 6 months, and 12.4% at 12 months. In contrast, TSH target achievement with thyroid hormone therapy increased over time, reaching 100% at 12 months. Patients with spontaneous normalization at 12 months were younger, had higher preoperative fT3, and lower preoperative TSH (all *P* < 0.05). Preoperative TSH was an independent predictor, with an optimal cutoff of ≤ 1.7 μIU/mL for predicting TSH ≤ 2 μIU/mL at 12 months.

**Conclusion:**

Approximately 12.4% of low-risk PTMC patients achieved spontaneous TSH normalization within one year after lobectomy, obviating hormone replacement. A preoperative TSH ≤ 1.7 μIU/mL serves as a useful predictor, supporting individualized management by deferring or avoiding levothyroxine supplementation in eligible patients.

## 1 Introduction

Thyroid cancer represents the most rapidly increasing endocrine malignancy worldwide. Global epidemiological data indicate approximately 586,000 new cases annually, representing the vast majority of endocrine-related cancers. Of all thyroid malignancies, papillary thyroid carcinoma (PTC) comprises about 85%−90% (1, 2).

Thyroid-stimulating hormone (TSH) suppression therapy remains a standard component of postoperative management for differentiated thyroid carcinoma (DTC). Experimental evidence shows that TSH stimulates normal thyrocytes and PTC cells via the TSH receptor, potentially inducing. Hyperplasia in residual thyroid tissue and neoplastic foci, thereby increasing recurrence risk (3, 4). Consequently, conventional management relies on exogenous levothyroxine (L-T4) administration to achieve TSH suppression, often at supraphysiological doses depending on the patient's risk profile. Through negative feedback, this suppresses serum TSH to subnormal or undetectable levels and inhibits tumor cell proliferation (5, 6). According to the American Thyroid Association guidelines and China's 2023 Diagnosis and Treatment Guidelines for Thyroid Nodules and Differentiated Thyroid Cancer (7, 8),routine TSH suppression is not advised for patients with low-risk PTC. Instead, maintaining serum TSH between 0.5 and 2.0 μIU/mL is considered adequate.

Papillary thyroid microcarcinoma (PTMC), which is defined as PTC with a maximum diameter ≤ 1.0 cm, offers three evidence-based management options for low-risk patients: active surveillance, radiofrequency ablation (RFA), and surgical intervention. While lobectomy remains the preferred surgical approach, emerging evidence suggests that long-term thyroid hormone replacement may be associated with multisystem morbidity and impaired quality of life. Potential concerns include neurocognitive impairments (e.g., attention deficit, memory decline), chronic fatigue, sleep disturbances, and psychological comorbidities, such as anxiety and depression. Furthermore, postoperative complications, often exacerbated by fluctuations in hormone levels, can manifest as somatic dysfunctions, including voice alteration and paresthesia (9, 10). There is a noticeable gap between patient concerns and clinical decision-making with respect to TSH suppression therapy. Patients commonly fear tumor progression and the risk of postoperative medication. In contrast, studies on clinical decision-making reveal that physicians maintain conservative stances: 48.8% would likely/very likely recommend TSH suppression for patients with low-risk PTC, whereas 29.7% extend this recommendation to very low-risk cases (11). This difference in risk assessment and expectations highlights the urgent necessity to improve postoperative management strategies for PTC. Given this context, our study targeted patients with low-risk PTMC who underwent unilateral lobectomy. Following currently prevalent guidelines, we aimed to: (1) Quantify the incidence of spontaneous TSH normalization (≤ 2 μIU/mL) at 0.5, 1.5, 6, and 12 months postoperatively; (2) Identify preoperative predictors of spontaneous TSH normalization. Using a predictive model based on preoperative characteristics, we hoped to set individualized criteria to safely omit TSH suppression therapy. This precision medicine approach aimed to connect patient-centered care and evidence-based practice in low-risk PTMC treatment.

## 2 Materials and methods

### 2.1 Definition of low-recurrence-risk stratification and cohort selection

Low-risk stratification was defined as intrathyroidal PTC meeting all the following criteria: absence of extrathyroidal extension, no vascular invasion, no clinical lymph node metastasis, or presence of ≤ 5 pathological N1 micro metastases (maximum diameter < 2 mm) (7). From November 2021 to June 2023, 617 adult patients with PTMC with lymph node-negative status underwent unilateral lobectomy at the Fifth Affiliated Hospital of Wenzhou Medical University (Li Shui Central Hospital). These patients were initially screened, and the diagnosis of PTMC was confirmed histologically using postoperative pathology. The study's exclusion criteria were: (1) Preoperative hypothyroidism or ongoing thyroid hormone replacement therapy; (2) Preoperative diagnosis of hyperthyroidism; (3) History of prior thyroid surgery; (4) Immediate postoperative thyroid hormone supplementation; (5) Lack of preoperative clinical data; (6) Follow-up duration < 12 months; (7) Previous radiotherapy or other oncologic treatments. After rigorous screening, 411 patients remained in the final cohort.

The study protocol was approved by the Institutional Ethics Committee of our center (Approval No.: 2025(I)-101-01).

### 2.2 Postoperative TSH monitoring protocol and outcome definitions

Patients undergoing unilateral lobectomy for PTMC were scheduled for serial thyroid function tests (TFTs) at 0.5, 1.5, 6, and 12 months postoperatively. Clinical management was dictated by the initial TFT at 0.5 months. When TSH > 2 μIU/mL, L-T4 supplementation was initiated immediately after the first test; When TSH ≤ 2 μIU/mL, L-T4 was withheld, but the TFT schedules continued as planned. At each follow-up, patients were stratified by TSH levels (> 2 or ≤ 2 μIU/mL). Key metrics were: 1) Spontaneous normalization rate: the percentage of unmedicated patients maintaining TSH ≤ 2 μIU/mL. 2) Pharmacologically achieved normalization rate: the percentage of patients reaching the ≤ 2 μIU/mL target following L-T4 supplementation.

### 2.3 Data collection and variable definitions

We retrospectively collected the following parameters from patients with low-risk PTMC: (1) Baseline clinical characteristics: sex, age, and body mass index (BMI); (2) Preoperative biochemical profiles: serum free triiodothyronine (fT3), free thyroxine (fT4), TSH, thyroid peroxidase antibody (TPO Ab), thyroglobulin (Tg), and thyroglobulin antibody (Tg Ab); (3) Histopathological features: tumor location, maximum diameter, multifocality, lymph node metastasis status, and concomitant thyroiditis.

### 2.4 Recurrence assessment

We evaluated recurrence of disease 12 months after surgery. Recurrence was defined as the detection of suspicious lymph nodes on cervical ultrasonography with at least two features of malignancy (short-axis diameter > 8 mm, loss of fatty hilum, rounded morphology, or abnormal vascularization). Cervical ultrasonography was performed at 6 and 12 months postoperatively. Malignancy was confirmed cytopathologically by ultrasound-guided fine-needle aspiration (FNA) using the 2023 Chinese Consensus on FNA Diagnostic Criteria. In patients with confirmed recurrence, therapeutic lateral neck dissection was performed to remove cervical lymph nodes from levels II–V.

### 2.5 Statistical analysis

Continuous variables were reported as the mean ± standard deviation for normally distributed data, or as the median and interquartile range (IQR) for non-normally distributed data. Intergroup comparisons were performed using the Student's t-test for normally distributed variables with equal variance, or the Mann–Whitney U test for skewed distributions. Categorical data were presented as frequencies and percentages, analyzed by Chi-square test or Fisher's exact test, as appropriate. Binary logistic regression was employed to identify preoperative predictors of spontaneous TSH normalization (i.e., postoperative TSH ≤ 2 μIU/mL without pharmacologic intervention). Predictive performance was evaluated using ROC curves, with the optimal cutoff value of preoperative TSH determined by maximizing the Youden index (sensitivity + specificity −1). The area under the curve (AUC) with its corresponding 95% confidence interval (CI) was reported to assess model discrimination. Statistical significance was defined as *P* < 0.05. All analyses were conducted using SPSS 22.0.

## 3 Results

**Figure 1:**
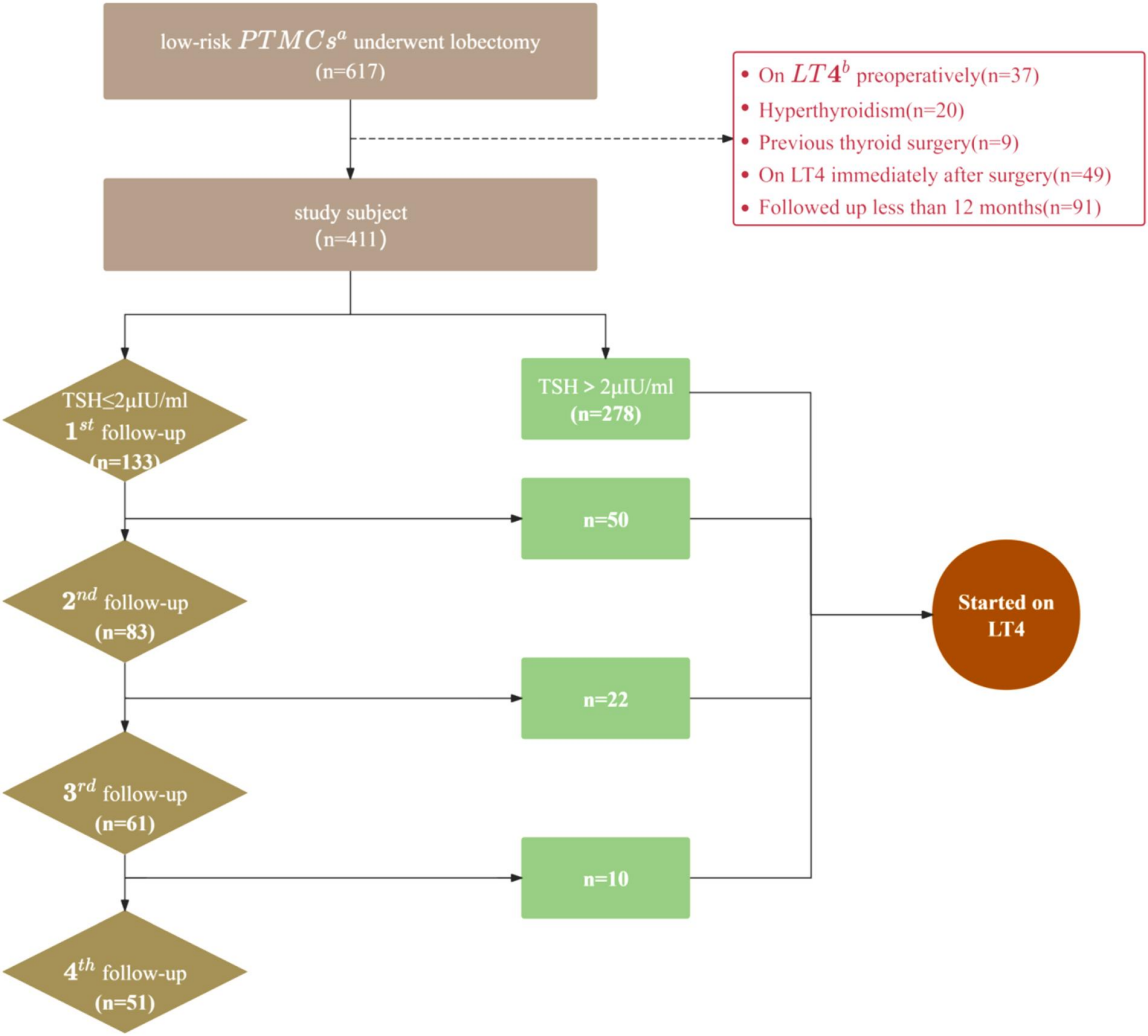
Study design. ^a^papillary thyroid microcarcinoma. ^b^Levothyroxine.

A total of 411 patients with low-risk PTMC met the inclusion criteria (Figure 1). Demographic and clinicopathological characteristics are summarized in Table 1. The cohort had a mean age of 47.7 ± 10.5 years, and 80.1% (329/411) of the participants were female. The median fT4 of patients was 0.97 ng/dL (IQR: 0.90–1.04) and the median serum TSH level was 1.73 μIU/mL (IQR: 1.23–2.60). There were 72 cases (17.5%) positive for TPO antibody and 124 cases (30.2%) positive for Tg antibody. Surgical pathology revealed a mean primary tumor size of 5.02 ± 2.10 mm. Lymphocytic thyroiditis was present in 23.6% (97/411) of patients, and multifocality was observed in 14.4% (59/411) of patients. Left radical lobectomy was performed in 51.8% (213/411) of cases, while right radical lobectomy was accounted for 48.2% (198/411).

**Table 1:** Baseline clinical characteristics of all patients.

Variables	Total (*n* = 411)
Age, years, Mean ± SD	47.66 ± 10.51
Sex, *n* (%)	
Female	329 (80.05)
Male	82 (19.95)
BMI, kg/m^2,Mean ± SD	24.30 ± 3.52
Preoperative fT3, pg/mL, Mean ± SD	2.72 ± 0.47
Preoperative fT4, ng/dL, median (IQR)	0.97 (0.90–1.04)
Tg, ng/mL, median (IQR)	10.38 (5.39- 16.58)
Preoperative TSH, μIU/mL, median (IQR)	1.73 (1.23–2.60)
Serum TPO antibody positive^a^, *n* (%)	
Yes	72 (17.52)
No	339 (82.48)
Serum Tg antibody positive^b^, *n* (%)	
Yes	124 (30.17)
No	287 (69.83)
Tumor size, mm, Mean ± SD	5.02 ± 2.10
Presence of lymphocytic thyroiditis, *n* (%)	
Yes	97 (23.60)
No	314 (76.40)
Multifocality	
Yes	59 (14.36)
No	352 (85.64)
Side of lobectomy	
Left	213 (51.82)
Right	198 (48.18)

BMI, body mass index; fT3, free triiodothyronine; fT4, free thyroxine; IQR, interquartile range; Tg, thyroglobulin; TSH, thyroid-stimulating hormone; TPO, thyroid peroxidase.

a Refers to values > 9IU/mL.

b Refers to values > 4IU/mL.

### 3.1 Temporal trends in postoperative TSH normalization

**Figure 2:**
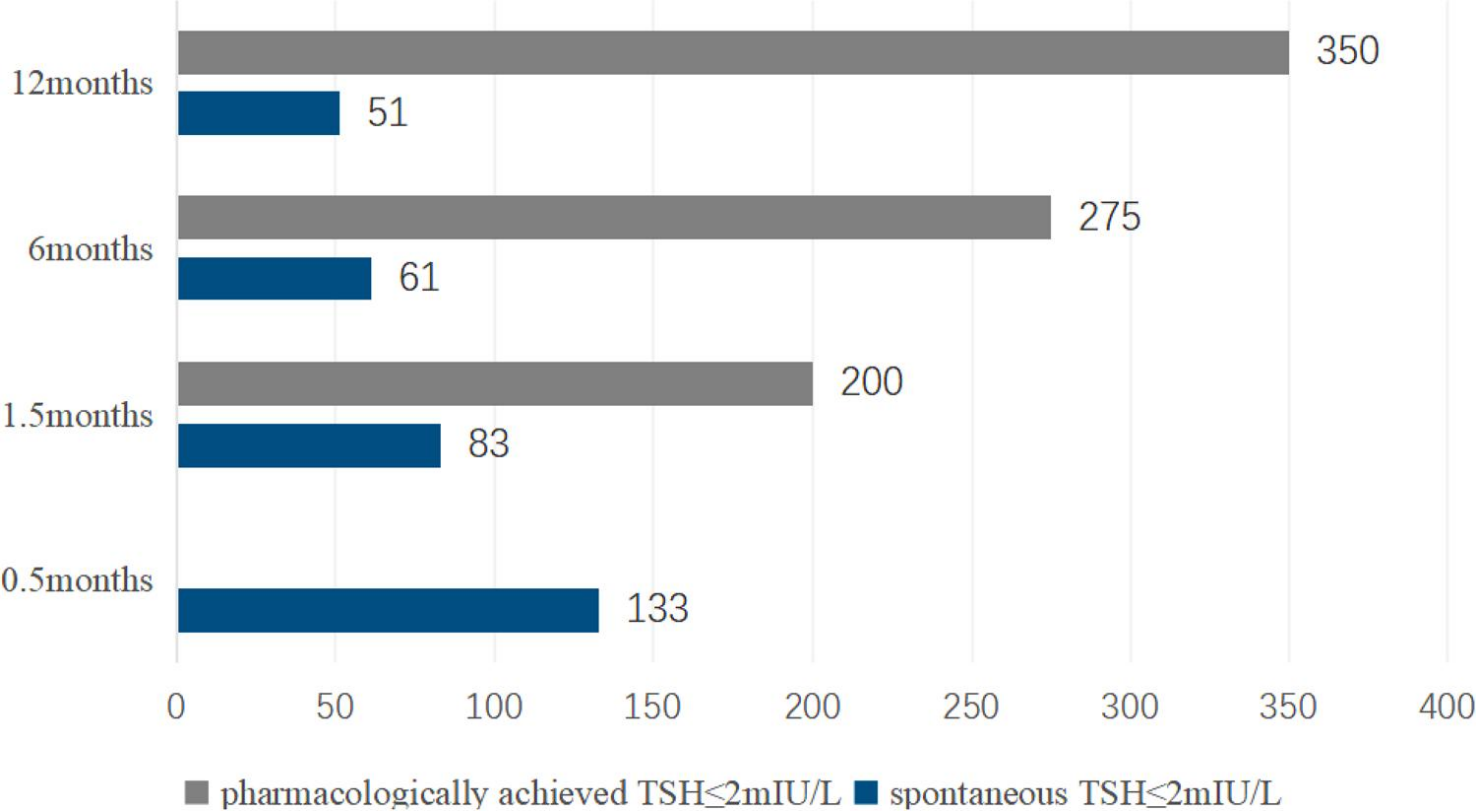
The proportions of patients with TSH ≤ 2 μIU/mL at 0.5, 1.5, 6, and 12 months after thyroid lobectomy.

The proportion of patients achieving spontaneous TSH normalization decreased over the course of follow-up: 32.4% (133/411) at 0.5 months postoperatively; 20.2% (83/411) at 1.5 months; 14.8% (61/411) at 6 months; and 12.4% (51/411) at 12 months (Figure 2). The rate of pharmacologically achieved normalization progressively increased over time, reaching 71.9% (200/278) at 1.5 months and 83.8% (275/328) at 6 months, ultimately achieving 100%(350/350) at 12 months. Among the 10 patients whose TSH levels remained > 2 μIU/mL at 12 months, subsequent L-T4 dose adjustments resulted in a 100% (10/10) normalization rate by 18 months.

### 3.2 Clinical predictors of spontaneous TSH normalization

At 12 months post-lobectomy, spontaneous normalization was significantly associated with younger mean age (44.92 ± 11.81 vs. 48.04 ± 10.27 years, *P* = 0.047), higher preoperative fT3 (2.85 ± 0.85 vs. 2.70 ± 0.38 pg/mL, *P* = 0.029), and lower preoperative TSH (median 1.09 μIU/mL (IQR: 0.70–1.44)vs. 1.90 μIU/mL (IQR: 1.31–2.77), *P* < 0.001). In the final multivariable model, preoperative TSH and fT3 remained independent predictors of spontaneous normalization (Tables 2, 3).

**Table 2:** Results of univariate analysis for clinical factors associated with TSH ≤ 2 μIU/mL and > 2 μIU/mL at 12 months after thyroid lobectomy.

Variables	TSH > 2 μIU/mL (*n* = 360)	TSH ≤ 2 μIU/mL (*n* = 51)	*t/*2*χ*	*P*
Age, years, Mean ± SD	48.04 ± 10.27	44.92 ± 11.81	1.993	0.047
Sex, *n* (%)			3.263	0.071
Female	293 (81.39)	36 (70.59)		
Male	67 (18.61)	15 (29.41)		
BMI, kg/m^2,Mean ± SD	24.29 ± 3.46	24.42 ± 3.92	0.453	0.651
Preoperative fT3, pg/mL, Mean ± SD	2.70 ± 0.38	2.85 ± 0.85	2.185	0.029
Preoperative fT4, ng/dL, median (IQR)	0.96 (0.89–1.03)	1.00 (0.94–1.06)	1.541	0.12
Tg, ng/mL, median (IQR)	9.99 (5.23–16.75)	11.05 (6.66–14.58)	0.727	0.468
Preoperative TSH, μIU/mL, median (IQR)	1.90 (1.31–2.77)	1.09 (0.70–1.44)	5.228	< .001
Serum TPO antibody positive^a^, *n* (%)			1.334	0.248
Yes	66 (18.33)	6 (11.76)		
No	294 (81.67)	45 (88.34)		
Serum Tg antibody positive^b^, *n* (%)			3.083	0.079
Yes	114 (31.67)	10 (19.61)		
No	246 (68.33)	41 (80.39)		
Tumor size, mm, Mean ± SD	5.06 ± 2.10	4.73 ± 2.10	2.386	0.122
Presence of lymphocytic thyroiditis, *n* (%)			3.149	0.076
Yes	90 (25.00)	7 (13.73)		
No	270 (75.00)	44 (86.27)		
Multifocality			0.084	0.772
Yes	51 (14.17)	8 (15.69)		
No	309 (85.83)	43 (84.31)		
Side of lobectomy			0.221	0.638
Left	185 (51.39)	28 (54.90)		
Right	175 (48.61)	23 (45.10)		

a Refers to values > 9IU/mL.

b Refers to values > 4IU/mL.

**Table 3:** Results of multivariate analysis for clinical factors associated with TSH ≤ 2 μIU/mL at 12 months after thyroid lobectomy.

Indicators	B	S.E.	Wals	*P*	OR	95% C.I.
preTSH	−2.571	.406	40.149	.000	.076	0.034–0.169
prefT3	.656	.301	4.743	.029	1.926	1.068–3.475

**Figure 3:**
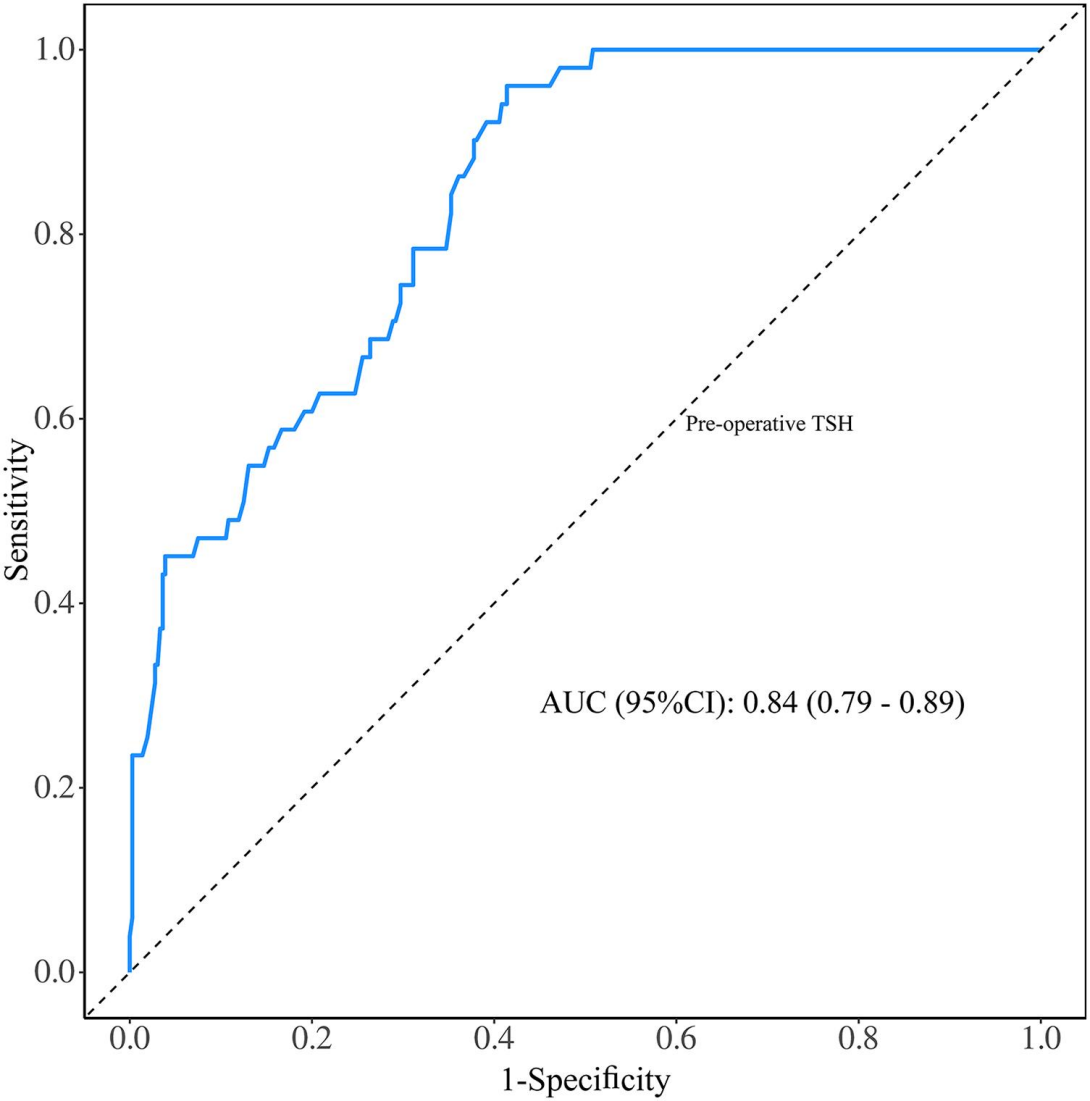
Receiver operating characteristic curve of the preoperative thyroid stimulating hormone (TSH) for the prediction of development of postoperative TSH > 2 μIU/mL. The optimal cut-off was settled at 1.7 μIU/mL (area under the curve = 0.84; *p* < .001).

A ROC curve was constructed to evaluate the predictive value of preoperative TSH levels for spontaneous normalization (TSH ≤ 2 μIU/mL) at 12 months postoperatively. Based on the maximum Youden index, the optimal preoperative TSH cutoff was 1.7 μIU/mL (sensitivity: 96%; specificity: 59%; AUC: 0.84; 95% CI: 0.80–0.87; Figure 3).

### 3.3 Postoperative recurrence surveillance

At 12 months post-lobectomy, cervical ultrasonography showed no evidence of recurrence in the thyroid bed or cervical lymph nodes in any patient. However, one patient on regular levothyroxine therapy was found to have a suspicious malignant nodule in the contralateral thyroid lobe at 12 months. FNA cytology confirmed PTC, prompting completion thyroidectomy and central neck dissection.

## 4 Discussion

In this single-center study of 411 adults with low-risk PTMC who underwent unilateral thyroid lobectomy, the rate of spontaneous TSH normalization progressively decreased over the 12-month follow-up, reaching 12.4% at the final time point. Two recent studies reported that 84%−90% of patients with low-risk PTC maintained TSH > 2 μIU/mL postoperatively (12, 13). However, data regarding pharmacologically achieved TSH normalization rates remain scarce in the current literature. For example, patients treated with L-T4, Zhang et al. reported that only 30% (80/268) achieved TSH < 2 μIU/mL at 12 months (14). In another multicenter cohort, pharmacologically achieved normalization was observed in 68.6% of with lobectomy patients at 12-month follow-up (15). Notably, these studies included intermediate/high-risk patients who required stricter TSH targets (< 0.5 or even < 0.1 μIU/mL), which might confound direct comparisons. By contrast, our cohort included only patients with low-risk PTMC with a TSH target ≤ 2 μIU/mL, who achieved a 100% pharmacologically achieved normalization rate at 12 months after L-T4 dose titration.

Current evidence indicates that 11% to 64% of patients undergoing unilateral thyroid lobectomy for PTC develop postoperative hypothyroidism, necessitating exogenous L-T4 supplementation to maintain euthyroidism (16–20). Research has demonstrated that preoperative elevated TSH levels and coexistent thyroiditis predict postoperative hypothyroidism (16–23). In our study, we identified age, preoperative fT3, and preoperative TSH as factors associated with postoperative TSH ≤ 2 μIU/mL, and multivariatble logistic regression confirming preoperative fT3 and preoperative TSH as independent predictors. Specifically, a preoperative TSH threshold of ≤ 1.7 μIU/mL predicted postoperative TSH ≤ 2 μIU/mL. Clinically, higher preoperative fT3 and lower TSH indicate robust thyroid reserve, suggesting a decreased requirement for exogenous hormone supplementation after lobectomy. Mao et al. reported that a greater tumor burden correlated with higher TSH levels; specifically, the mean tumor size was significantly larger in the highest TSH quartile compared to the lowest (6.6 ± 2.22 mm vs. 6.1 ± 2.54 mm; *P* = 0.0028) (24). However, contrary to previous studies, we found no significant correlation between chronic lymphocytic thyroiditis (CLT)/TPO Ab positivity and postoperative TSH levels (*P* > 0.05). This discrepancy may reflect the inclusion of patients with ultrasound-suspected chronic CLT in earlier studies. An extended follow-up may elucidate CLT's impact on residual thyroid functional.

In addition to these patient-specific and tumor-specific predictors, the regulation of thyroid function itself is increasingly understood to be modulated by systemic factors, with inflammation being a key player. Emerging research suggests that systemic inflammatory status, beyond local autoimmune thyroiditis, may independently influence thyroid physiology. For instance, a large cross-sectional study based on the US NHANES population found that the Systemic Inflammatory Response Index (SIRI) was significantly positively correlated with serum levels of free thyroxine (FT4), total thyroxine (TT4), and thyroid peroxidase antibodies (TPOAb) (25). This indicates that a higher systemic inflammatory burden is associated with higher thyroid hormone levels and a stronger autoimmune response, providing a broader perspective for understanding thyroid regulation. It suggests that in the long-term management of thyroid function after lobectomy, a patient's systemic inflammation level could be a potential confounding variable worthy of future investigation to distinguish its role from that of local inflammation.

Importantly, the clinical significance of maintaining euthyroidism extends far beyond local or systemic inflammatory interactions, as thyroid dysfunction itself exerts profound systemic consequences. Baykal emphasized that normal thyroid function is a conditio sine qua nonfor reproductive health, with dysfunctions directly causing menstrual disorders, infertility, adverse pregnancy outcomes, and impaired fetal neurodevelopment (26). This systemic impact further underscores the importance of balancing TSH control and quality of life in the long-term management of low-risk PTMC patients.

In strict accordance with current guidelines, we initiated L-T4 supplementation upon detecting TSH levels > 2.0 μIU/mL during follow-up. Nevertheless, it is important to recognize that an elevation in TSH following unilateral lobectomy may be a transient compensatory response during the early postoperative stage, with the potential for subsequent spontaneous normalization. For example, in a study by Xiao et al. (12) among 115 patients with DTC who underwent unilateral lobectomy, 97 (84.3%) had TSH > 2 μIU/mL over a median follow-up period of 2.6 years, while 18 (15.7%) had TSH ≤ 2 μIU/mL. Notably, five patients demonstrated spontaneous normalization (TSH ≤ 2 μIU/mL) without any intervention 9 months after surgery. These findings suggest that our protocol might have overlooked such opportunities for spontaneous TSH normalization. Therefore, to address uncertainties, larger prospective cohorts are required to clarify the dynamic TSH trajectory in patients with PTMC after lobectomy and to optimize the timing of initiating exogenous thyroid hormone supplementation.

Previous studies indicated that TSH suppression therapy can reduce recurrence rates by 25% and cancer-specific mortality by 50% (4). Nevertheless, recent evidence has cast doubt on its protective effect in low-risk patients with PTC (27, 28). At the same time, it's important to weigh these findings against known risks: long-term supraphysiological TSH suppression raises the risks of cardiovascular morbidity (particularly atrial fibrillation and cardiac remodeling), osteoporosis, and secondary cancer incidence (5, 29). In a cohort of low-risk patients with PTMC who underwent unilateral lobectomy (17), only three cases (0.8%) exhibited recurrence within the first year: two with *de novo* central lymph node metastasis and one with surgical bed recurrence, with no reported cancer-related mortality. Similarly, in our cohort, no recurrences at the surgical bed, nodal metastases, or cancer-specific mortality were observed during the 12-month follow-up period. Adding to this evidence, a landmark 2024 study retrospectively analyzed 11,140 patients with PTC (median follow-up 70 months), demonstrating no correlation between postoperative TSH suppression and tumor recurrence (HR 1.24, 95% CI 0.81–1.91) (30). Critically, lower TSH levels conferred no prognostic advantage–the RFS rates at 5/10 years were identical across TSH strata (≤ 0.5 vs. > 3 μIU/mL: 97.8% vs. 97.3%; *P* = 0.83). Thus, mildly elevated TSH may be considered safe for low-risk PTMC after lobectomy. Further supporting this, Sugitani's randomized trial, which primarily included low-risk patients according to the Age, Metastasis, Extent and Size criteria, revealed no statistically significant difference in recurrence (*P* > 0.05) or disease-free survival between the strict TSH suppression group and the euthyroidism-maintained group over a 6.9-year follow-up (31). In alignment with these observations, the 2024 Japanese Clinical Guidelines for Thyroid Tumor Management explicitly state that patients who have undergone thyroid lobectomy do not require routine TSH suppression therapy (32). Taken together, and in light of the evolving trends in domestic and international guidelines, the TSH control targets for patients with low recurrence risk are being gradually relaxed. This shift indicates that TSH suppression treatment and monitoring are evolving from a focus on target achievement, active prevention, and treatment of side effects, to a more individualized, precise, and dynamic assessment approach. If future research further refines the optimal TSH target for low-risk patients unilateral lobectomy, a significant and increasing number of patients may be spared the necessity of long-term TSH suppression therapy. Nevertheless, to further validate these shifts in treatment strategies, multicenter prospective studies are necessary to more definitely verify the relationship between exogenous thyroid hormone and the long-term risk of disease progression and recurrence.

Current management strategies for low-risk PTMC include surgery, thermal ablation, and active surveillance. For example, a recent retrospective study (33), comparing thermal ablation (*n* = 229) with surgery (*n* = 453) in patients with cT1N0M0 PTC revealed no significant difference in overall survival after propensity score matching (median follow-up: 20 vs. 26 months). Notably, 35.5% (161/453) of the surgical patients developed lymph node metastases (median metastatic foci: 3; IQR 2–4), indicating a similarly high prevalence of occult nodal involvement among candidates for ablation. These findings underscore the unpredictability of long-term oncologic outcomes associated with non-surgical approaches. Regarding active surveillance, data from a Korean cohort (34) demonstrated that over a median follow-up of 32.5 months, tumor volume progression occurred in 23.2% (86/371) of patients, while significant and diameter enlargement was observed in 3.5% (13/371). The cumulative incidence of progression escalated over time (2/3/4/5year rates: 6.9%, 17.3%, 28.2%, and 36.2%, respectively), a trend that correlated with heightened patient anxiety and fear of disease advancement. Therefore, based on our cohort data, surgical resection may serve as a more proactive therapeutic strategy for low-risk patients with PTMC who experience persistent cancer-related psychological distress or are unable to tolerate postoperative pharmacotherapy. This is especially relevant when preoperative TSH levels are ≤ 1.7 μIU/mL–which we identified as a significant predictor of spontaneous TSH normalization following lobectomy. For these individuals, initial postoperative management via active surveillance without immediate TSH suppression constitutes a clinically sound and viable alternative.

## 5 Conclusion

In conclusion,12.4% of the patients with low-risk PTMC who underwent unilateral lobectomy achieved and maintained a serum TSH ≤ 2 μIU/mL without pharmacological intervention at the 12-month follow-up mark. A lower preoperative TSH level serves as a strong independent predictor of this outcome, with a threshold of ≤ 1.7 μIU/mL identifying patients most likely to maintain euthyroidism spontaneously. This finding offers evidence to support an individualized exemption from TSH suppression therapy and provides a framework for personalized preoperative counseling regarding postoperative management strategies.

Our study has several limitations that warrant acknowledgment. First, as a retrospective cohort analysis, it is inherently susceptible to selection bias and unmeasured confounding factors. Second, the sample size (*n* = 411) was relatively modest, and this was a single-center retrospective study including only node-negative patients with PTMC who underwent unilateral lobectomy. Consequently, the generalizability of our findings to patients undergoing lobectomy for other indications, such as benign thyroid nodules or intermediate-risk carcinomas, remains limited.Future multi-center, large-scale prospective studies are warranted to validate our results and improve external validity. In addition, combining radiomic features from medical imaging may help identify more comprehensive predictive indicators and further clarify the influencing factors of postoperative TSH normalization. We will also incorporate radiomic analysis in subsequent research to build more accurate predictive models and strengthen the clinical evidence base.

## Data Availability

The original contributions presented in the study are included in the article/Supplementary Material, further inquiries can be directed to the corresponding authors.
